# Are Primo Vessels (PVs) on the Surface of Gastrointestine Involved in Regulation of Gastric Motility Induced by Stimulating Acupoints ST36 or CV12?

**DOI:** 10.1155/2012/787683

**Published:** 2012-10-04

**Authors:** Xiaoyu Wang, Hong Shi, Hongyan Shang, Yangshuai Su, Juanjuan Xin, Wei He, Xianghong Jing, Bing Zhu

**Affiliations:** ^1^Institute of Acupuncture and Moxibustion, China Academy of Chinese Medical Sciences, Beijing 100700, China; ^2^Shandong University of Traditional Chinese Medicine, Jinan 250355, China

## Abstract

Previous studies showed primo vessels (PVs), which were referred to as Bonhan ducts (BHDs) and a part of circulatory system by Kim, located in different places of the body. The BHDs system was once considered as the anatomical basis of classical acupuncture meridian but not clearly identified by other investigators. In the present study, we tried to address the relationship between PVs and meridians through detecting the modulation of gastric motility by stimulating the PVs on the surface of stomach or intestine, as well as acupoints Zusanli (ST36) and Zhongwan (CV12). The results showed electric stimulation of the PVs had no effect on the gastric motility. While stimulating CV12 inhibited gastric motility significantly in PVs-intact and PVs-cut rats, there is no significant difference between the inhibition rate of the PVS-intact and the PVS-cut rats. Stimulating at ST36 increased gastric motility significantly in both the PVs-intact and the PVs-cut rats, yet there was no significant difference between the facilitation rate of the both groups. Taken together, the PVs on the surface of stomach or intestine did not mediate the regulation of gastric motility induced by stimulating at the acupoints ST36 or CV12.

## 1. Introduction

Acupuncture, as an important part of traditional Chinese medicine, is believed to restore the balance of *qi* and blood, as well as yin and yang, regulates functions of the viscera by dredging meridians. The meridian system plays a pivotal role in regulation and maintenance of physiological functions, and mediation of effects of acupuncture and moxibustion on visceral organs. So far there are many hypotheses explaining the mechanism and phenomena of acupuncture or meridian. Many researchers in this field tend to believe that the scientific bases of meridian and acupuncture can be explained by neurophysiological theories [1–3]. Langevin and Yandow and Langevin et al. proposed that acupoints and meridians were parts of a network formed by interstitial connective tissues [4, 5]. Another study provided evidence that the liquid-crystal collagen fibers that formed the bulk of connective tissue may conduct sound or electricity [6]. In addition, Zhang et al. proposed the low hydraulic resistance channel theory [7]. However, the anatomical structures of meridians still remain elusive.

Recently a series of studies from Prof. Soh KS reported that the primo vessels (PVs) are located in different places of the body, like the surface of the internal organs of rats, rabbits, and swine [8–10], inside the blood and lymphatic vessels [11, 12], in the epineurium, running along the sciatic nerve [13], and below the skin [14]. These PVs, also referred to as Bonhan ducts (BHDs), were part of a circulatory system that was first reported by Kim [15, 16] in the early 1960s. The BHDs system, which included several subsystems, was once considered as the anatomical basis of classical acupuncture meridians. The structure was also found by Fujiwara's followup [17]. Unfortunately, Bonghan theory was not clearly confirmed by most investigators [18]. The main reason is that the method employed by Kim was not disclosed, and the experiments were hard to reproduce. In addition, nobody addressed the role of BHD in the effect of acupuncture. The structure of the PVs is distinct from the well-known tissues, such as nerves, blood vessels, lymph-vessels, and blood capillaries. But there is still no research focusing on the relationship between the PVs and acupuncture meridian. In order to elucidate the relationship between the PVs and acupuncture meridians, the possible impacts of the PVs on the effectiveness of acupuncture should be investigated. 

Acupuncture has been widely used for treating functional gastrointestinal (GI) disorders [19]. Previous studies showed that acupuncture at the lower limbs (ST36) causes muscle contractions via the somatoparasympathetic pathway, while acupuncture at the upper abdomen (CV-12) causes muscle relaxation via the somatosympathetic pathway [20, 21]. Are the PVs on the surface of gastrointestine involved in regulating process of gastro motility induced by stimulating ST36 or CV12? In this paper, we detected the effects of stimulating the PVs on gastric motility and whether the PVs play a role in the regulation of gastric motility by stimulating ST36 or CV12, and tried to address the relationship between the PVs and meridians.

## 2. Materials and Methods

### 2.1. Animal Preparation and Methods for Identifying PVs

Thirty Sprague-Dawley (SD) rats (both sexes, 200–230 g) were purchased from Institute of Animal, Academy of Chinese Medical Sciences. The animals were housed under a 12 h light/dark with free access to food and water. All animals were treated according to the Guide for Use and Care of Medical Laboratory Animals from Ministry of Public Health of People's Republic of China.

 The animals were fasted overnight with free drinking of water in proxima luce and were anesthetized with urethane (1.5 g kg^−1^), and all surgical procedures were performed under anesthesia. About 1 h after the urethane administration, the rats were under deep anesthesia, and the trachea was cannulated but not immobilized to keep respiratory tract unobstructed and a catheter was inserted into one of the jugular veins for infusion. The abdominal sides of the rats were incised, and the identification of the PVs was carried out under a stereomicroscope (Nikon SMZ750) according the method reported by Lee et al. [22]. The images of the PVs on the organ surfaces were recorded using a CCD camera in situ and in vivo (Nikon SMZ750).

When the PVs were located, 0.2% diluted Trypan blue solution 1-2 mL was dropped to stain them on the internal organs such as the stomach, the small intestine, the large intestine, and the urinary bladder and the wall of the peritoneum of the rat for 20–30 sec, and then the internal organs were washed for several times with 10 mL of phosphate-buffered saline, PH 7.4 (PBS) for detection of the PVs. Under a stereomicroscope with a CCD camera (Nikon SMZ750), the stained PVs were observed, and the images were taken.

### 2.2. Gastric Motility Recording

After identifying the PVs, gastric motility were recorded. A small longitudinal incision was made in the duodenum about 2-3 cm from the pylorus. A small balloon made of flexible condom rubber was inserted via incision of the duodenum into the pyloric area of rat and kept in position by tying the connecting catheter to the duodenum, and another catheter (inner diameter of 1 mm) was also inserted into the same hole by incision in order to drain digestive juices secreted from stomach. The balloon was filled with warm-water about 0.2–0.5 mL, which gave about 80–150 mmH_2_O pressures. Pressure in the balloon was measured by a transducer (NeuroLog, NL900D) for low pressure through a thin polyethylene tube (1.5 mm in outer diameter) and then input on a polygraph (NeuroLog, NL900D) amplifier and led into a data acquisition system (Power-Lab) for further analysis. Demifasting gastric motor activity was recorded as a control for at least 1 h before acupuncture stimulation [20, 21].

The changes of gastric motility induced by the stimulation were compared with the basal activity recorded before any stimulation. If the change rates of gastric motility during stimulation were 15–20% of the basal activity, the response was then considered to have an excitatory or inhibitory regulation, respectively. Systemic blood pressure and heart rate were continuously monitored by using of Biopac data acquisition system (MP150, USA). Both signals were analyzed offline using the CED 1401-plus data system and the Spike 2 package (Cambridge Electronic Devices, Cambridge, UK), Rectal temperature was kept constantly around 37°C by a temperature controller (DC, USA).

### 2.3. Acupuncture and Electroacupuncture Stimulation of CV12 or ST36

A needle (0.3 mm in diameter) was inserted into the skin and its underlying muscles at acupoints Zhongwan (CV12) and Zusanli (ST36) on the body. CV12 was located at center of abdomen, in middle line of the body. ST36 was located bilaterally at the anterior tibia muscles near the knees. For the manual acupuncture (MA), the needle was rotated clockwise and anticlockwise at 2 Hz for 3 min. In electroacupuncture (EA) experiments, acupoints were stimulated by a pair of needle-electrodes inserted 0.3–0.5 cm deep into the skin, and electroacupuncture intensities were set as 5 mA, with frequency of 2/15 Hz alternatively for 3 min. Procedure of the experiments was performed as follows: (1) firstly manual acupuncture or electroacupuncture at ST36 or CV12, (2) then stimulated the PVs with different frequency, (3) manual acupuncture or electroacupuncture at ST36 or CV12 after PVs was cut. Besides the heart rate and the blood pressure, the gastric pressure should be kept stable before stimulating the acupoint. 

### 2.4. Electrostimulation of PVs in Different Intensities

Bipolar stainless-steel electrodes were placed at the PVs for stimulation. Stimuli were given at different intensities of 1–20 mA, increments 2 mA, duration 2 ms, and 20 Hz for 30 s. 

### 2.5. Statistical Analysis

The data obtained before and after treatment in the same group or different group was compared statistically by a paired *t*-test or unpaired *t*-test. *P* < 0.05 was considered as a statistical significance. All data are expressed as mean ± SE.

## 3. Results

### 3.1. Distribution of PVs on the Surface of the Internal Organs and the Amount

**Figure 1:**
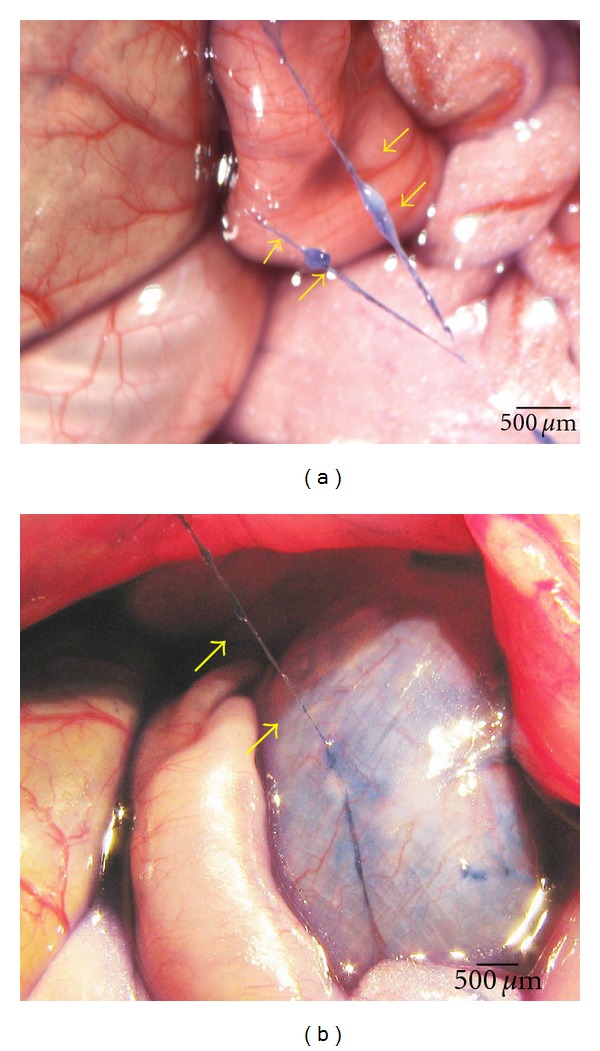
In situ and in vivo stereomicroscopic image of a typical primo vessel (arrow) and a corpuscle (arrow). (a) The primo vessel on the surface of intestine was stained with Trypan blue. (b) The primo vessel on the surface of stomach stained with Trypan blue. The primo vessels are semitransparent, freely movable strands irregularly fixed on the peritonea.

The PVs and the corpuscles were observed on surfaces of different internal organs, such as the stomach, liver, large and small intestines, and bladder.  Figure 1 showed a representative stereomicroscopic image of a PV (arrow) and its corpuscles (arrow) on the surface of the stomach and small intestine, respectively. The PVs observed in the present work are thin, semitransparent, freely movable strands that are randomly fixed on the peritonea, same as described by Lee et al. [22]. Among the total 30 rats, the PVS was observed in 23 rats. There is no difference between sexes. The percentage of the PVs emergence was 76.67%. There were 1 or 2 PVs observed on the surface of the gastrointestine in most rats, sometimes 1 PV was observed on the surface of the liver. 

### 3.2. PVs Cut Did Not Change the Inhibition of Gastric Motility Induced by CV12

The Gastric motility was detected in 23 rats by recording the intragastric pressure. Sometimes the gastric pressure did not recover to the baseline after stimulation of acupoints. Only two rats underwent all the manipulations, the other rats underwent two or more of the manipulations. When the intrapyloric balloon pressure was increased to about 80–200 mmH_2_O, the rhythmic waves of contractions in pyloric area were observed. With regard to gastric motor characteristics, it was noteworthy both the changes of intragastric pressure and rhythmic contraction. Generally, the intragastric pressure represents the index of gastric tone motility and rhythmic contraction represents gastric peristalsis induced by circular muscle contractions, similar to slow wave of gastric motor activity. The pressure was maintained at about 100 mmH_2_O as a baseline by expanding the volume of the balloon with warm water, rhythmic contractions occurred at a rate of four to six per minute, and these rhythmically gastric contractions could be recorded in both the PVS intact and PVs-cut rats.

**Figure 2:**
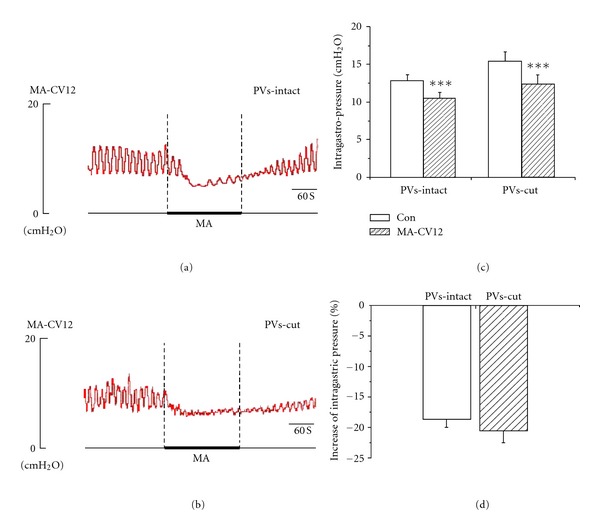
The response of gastric motility to the MA stimulation of CV12 in PVs-intact and PVs-cut rats. (a) and (b) Representative recording of gastric pressure during MA stimulation of CV12 before and after PVs-cut, respectively. (c) The inhibition of gastric motility induced by stimulating CV12 was of great significance in PVs-intact rats (*n* = 6, *P* < 0.01) and PVs-cut rats (*n* = 6, *P* < 0.01). (d) Comparing the inhibition effect between PVS-intact and PVS-cut, the inhibition rate was not significantly changed (*n* = 6, *P* > 0.05).

**Figure 3:**
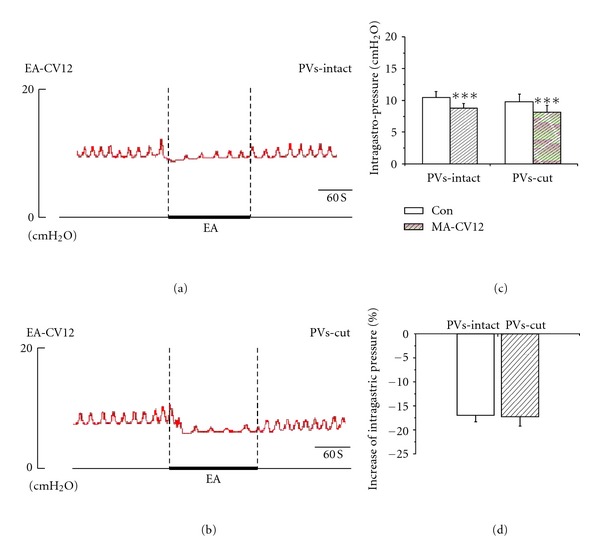
The response of gastric motility to the EA of CV12 in PVs-intact and PVs cut rats. (a) and (b) Representative recording of gastric pressure during EA stimulation of CV12 before and after PVs-cut, respectively. (c) The inhibition of gastric motility induced by stimulating CV12 was of great significance in PVs-intact rats (*n* = 9, *P* < 0.001) and PVs-cut rats (*n* = 9, *P* < 0.001). (d) Comparing the inhibition effect between PVS-intact and PVS-cut, the inhibition rate was not significantly changed (*n* = 9, *P* > 0.05).

MA and EA stimulation resulted in high suppression of gastric tonic motility with a rapid onset, followed by an obvious inhibition of rhythmic contraction waves, consistent with previous reports [20, 21]. These suppressions lasted throughout the period of acustimulation and even lasted about 3 min after withdrawing the needles in most cases. As shown in Figures 2(a), 2(c), 3(a), and 3(c), the intragastric pressure decreased from 12.82 ± 0.83 mmH_2_O to 10.46 ± 0.79 mmH_2_O in MA of CV12, the inhibition rate was 18.66 ± 1.28%. For EA stimulation of CV12, the intragastric pressure decreased from 10.51 ± 0.86 mmH_2_O to 8.75 ± 0.77 mmH_2_O, the inhibition rate was 17.02 ± 1.31%. Compared to the basal pressure, both inhibition effects were of great significance (***P < 0.001). In order to detect the contribution of the PVs to the inhibition of gastric motility, we designed an experiment in which the PVs were removed and then the effects of MA and EA stimulation at CV12 on gastric motility were examined. As shown in Figures 2(b), 2(d), 3(b), and 3(d), the inhibition of CV12 was not changed significantly after the PVs was cut. The intragastric pressure decreased from 15.48 ± 1.22 mmH_2_O to 12.38 ± 1.20 mm H_2_O by MA CV12, the inhibition rate was 20.21 ± 1.89%. For EA CV12, the intragastric pressure decreased from 9.84 ± 1.16 mmH_2_O to 8.18 ± 1.06 mmH_2_O, the inhibition rate was 17.34 ± 1.86%. Both inhibition effects were of great significance (***P < 0.001). Regardless of MA or EA, the inhibition rate was not significantly changed after removing the PVs (*P* > 0.05). 

### 3.3. PVs Did Not Mediate the Facilitation of Gastric Motility Induced by ST36

**Figure 4:**
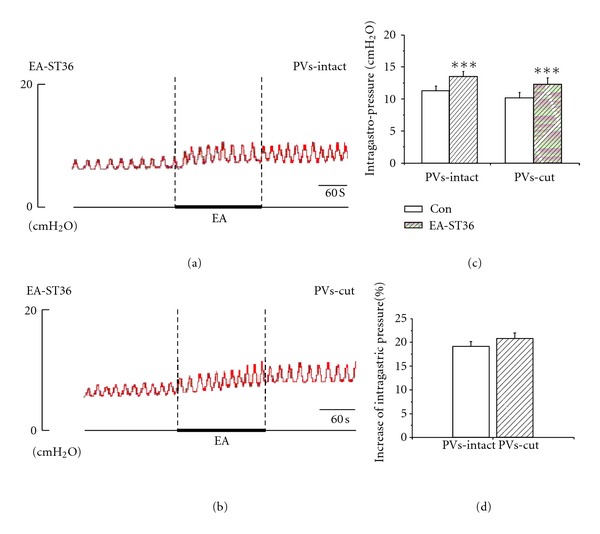
The response of gastric motility to the MA of ST36 in PVs-intact and PVs-cut rats. (a) and (b) Representative recording of gastric pressure during MA stimulating ST36 before and after PVs-cut, respectively. (c) The facilitation of gastric motility induced by stimulating ST36 was of great significance in PVs-intact rats (*n* = 5, *P* < 0.001) and PVs-cut rats (*n* = 5, *P* < 0.001). (d) Comparing the facilitation effect between PVS-intact and PVS-cut, the facilitation rate was not significantly changed (*n *= 5, *P* > 0.05).

**Figure 5:**
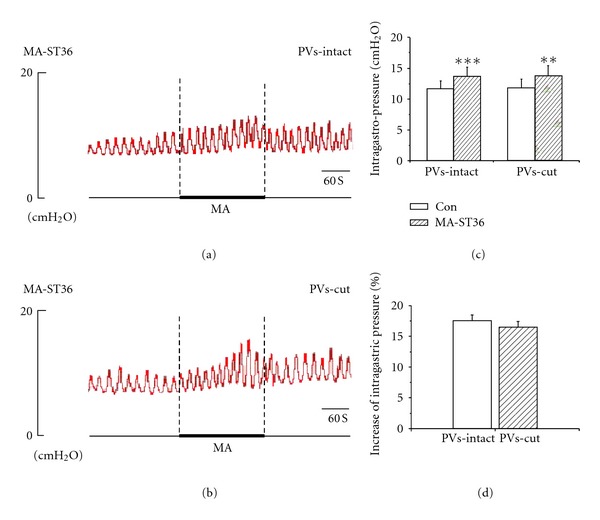
The response of gastric motility to the EA of ST36 in PVs-intact and PVs-cut rats. (a) and (b) Representative recording of intragastric pressure during MA stimulation of ST36 before and after PVs-cut, respectively. (c) The facilitation of gastric motility induced by stimulating ST36 was of great significance in PVs-intact rats (*n *= 9, *P* < 0.01) and PVs-cut rats (*n *= 9, *P* < 0.01). (d) Comparing the facilitation effect between PVS-intact and PVS-cut, the facilitation rate was not significantly changed (*n* = 9, *P* > 0.05).

MA and EA stimulation at ST36 caused a slight-moderate facilitation of gastric motility with a rapid onset and followed by a tonic motor that lasted throughout the period of acustimulation. The facilitation lasted throughout the period of acustimulation and even lasted about 1-2 min after withdrawing the needles in most cases. Figures 4(a), 4(c), 5(a), and 5(c), showed that both MA (*n* = 5) and EA (*n* = 9) at ST36 induced facilitation of gastric motility, consistent with the previous reports [20, 21]. The intragastric pressure increased from 11.63 ± 1.33 mmH_2_O to 13.64 ± 1.52 mmH_2_O in MA ST36, the facilitation rate was 17.47 ± 1.33%. For EA stimulation of ST36, the intragastric pressure increased from 11.34 ± 0.72 mmH_2_O to 13.47 ± 0.80 mmH_2_O, the facilitation rate was 19.13 ± 1.01%. Both reinforcement effects were of great significance (**P < 0.01, ***P < 0.001). We also examined the contribution of the PVs to the facilitation of gastric motility by removing the PVs here. Interestedly, as shown in Figures 4(b), 4(d), 5(b), and 5(d), the facilitation effect of needling ST36 did not changed after the PVs was cut, the intragastric pressure increased from 11.81 ± 1.41 mmH_2_O to 13.77 ± 1.67 mmH_2_O by MA stimulation at ST36 after removing the PVs, with a facilitation rate of 16.50 ± 0.51%. For EA ST36, the intragastric pressure increased from 10.23 ± 0.83 mmH_2_O to 12.32 ± 0.93 mmH_2_O after removing the PVs, and the facilitation rate was 20.79 ± 1.21%. Both facilitation effects were still of great significance (**P < 0.01, ***P < 0.01). The results suggested that removing PVs did not change the enhanced effects of gastric motility induced by ST36. 

### 3.4. Electrostimulation of PVs Did Not Affect the Changes of Gastric Motility Induced by ST36 or CV12

**Figure 6:**
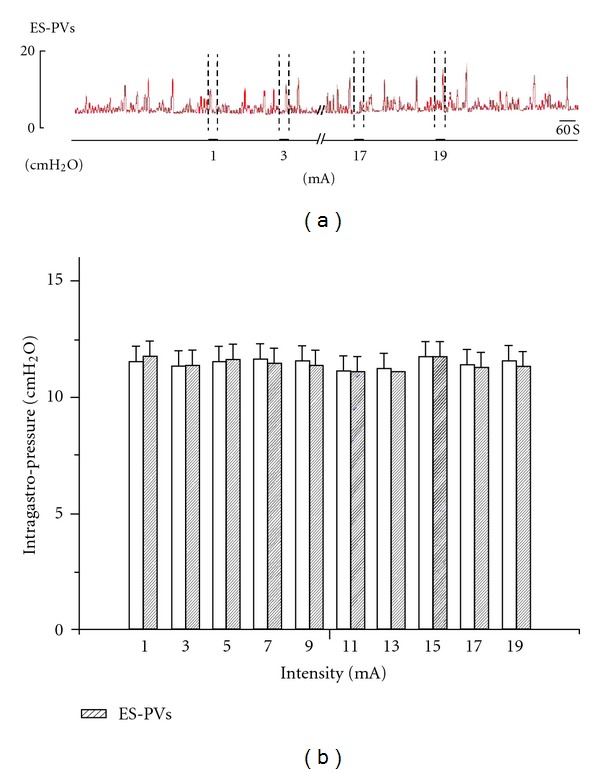
Electro-stimulation of PVs with different intensities by bipolar stainless-steel electrodes. Stimuli were given with different intensities from 1 to 19 mA, increments 2 mA, duration 2 ms, 20 Hz for 30 s. (a) A representative recording of the intragastric pressure under electro-stimulation of PVs with different intensities. (b) The intragastric pressure was not changed significantly by electro-stimulating PVs with different intensities from 1–19 mA (*n* = 11, *P* > 0.05).

From above, we knew that the PVs were not involved in the regulation of gastric motility by EA/MA at CV12 or ST36. A recent study [23] showed that the action potentials of the PVs had two types of pulses that are different from those of smooth muscle. Because action potentials occur in excitable cells, the electrophysiological characteristics of the PVs indicate that the PVs are excitable. To observe the changes of intragastric pressure induced by electro-stimulating the PVs with different intensities, here the bipolar stainless-steel electrodes were placed at the PVs for stimulus. As shown in Figure 6, the gastric pressure was not changed significantly by electrostimulating the PVs with different intensities from 1–19 mA (*P* > 0.05). The result indicated that the gastric motility was not affected by electrostimulating the PVs.

## 4. Discussion

In this study, we investigated the effects of stimulating the PVs on gastric motility in situ and in vivo, the role of PVs in the regulation of gastric motility by stimulating acupoints ST36 or CV12, and the relationship between the PVs and meridians. Stimulating the PVs with different intensities did not change the intragastric pressure in comparison with baseline. The results indicated that although the PVs were an excitable tissue [23], the PVs on the surface of internal organs did not show an effect on the regulation of gastric motility. To confirm whether the PVs in the internal organs play a role in the regulation of gastric motility by stimulating ST36 or CV12, the effects of acupuncturing at CV12 or ST36 on gastric motility were observed in PVs intact and PVs-removing rats. The results indicated that MA/EA-stimulating CV12 actually caused inhibition effect on gastric motility, otherwise MA/EA stimulating ST36 caused facilitation effect on gastric motility. This was in accordance with previous reports [24]. After the electric stimulation of the PVs was finished and the PVs were removed, the inhabitation and facilitation of gastric motility induced by acupuncturing CV12 or ST36 did not change significantly. These results showed that the PVs were not involved in the regulation of gastric motility by stimulation of CV12 or ST36. 

PVs, which were widely distributed and had a lot of characteristics and medical significance [25], are speculated to be involved in many processes of life, such as circulatory function, excitability, hormone path, immune function, hematopoiesis, regeneration and sanal, obesity and cancer, and so on. Therefore, the PVs may play endocrine and immunological roles, which might be involved in therapeutic effects of acupuncture. However, our current study provides evidence that the PVs were not essential for the regulation of gastric motility by stimulating CV12 or ST36. 

The anatomical structure of meridian had attracted many scientists since Dr. Kim declared that he found BHD and BHC in 1963 [15]. Some scientists taking part in the work recorded and narrated the process of the repeating experiments [18]. Combined with the restricted data at that time in China, the BHD was just found in a navel of an infant rabbit, including chromaffin cells, with smooth muscle outside. But when a rabbit grew up, the structure disappeared, suggesting that the structure of BHD is a degenerative tissue. Although the existence of the phenomena of meridians has been confirmed [26], the physical structure of meridian still remains controversial. Yet it is accepted by most researchers that there is relevance between the acupoints and internal organs. Different visceral diseases can be reflected in different acupoints. And acupuncture at different acupoints can regulate the functions of different visceral diseases. The meridians play important roles in the relevance between the acupoints and internal organs. So, in the present study, we try to investigate the relationship between the PVs and meridians by invesgating the relationship between the PVs and acupoints. Now the question is: although Soh et al.'s findings provide the evidence that the PVs exist in the internal organs, these findings did not address the function of the PVs, and thus are not enough to verify the relationship between the PVs and meridians. Our data also did not support an existence of the relationship between the PVs and the effects of acupuncture stimulation. In the future, we will have to further study the function of the PVs and their role in the regulation of visceral organs by acupuncture, and then elucidate the relationship between the PVs and the effects of acupuncture stimulation.

## 5. Conclusions

The PVs in surface of internal organs did not have an effect in regulating gastric motility. The PVs in the surface of internal organs were involved neither in the inhibition of the gastric motility induced by acupuncturing at CV12 nor in the facilitation of gastric motility induced by acupuncturing at ST36. Further research about the functional relationship between the PVs and meridian is needed in the future.
